# Rapid Whole Genome Sequencing Enhances Pediatric Cancer Diagnosis

**DOI:** 10.1038/s41467-026-77918-x

**Published:** 2026-10-06

**Authors:** Aditi Vedi, Jamie Trotman, Joao M. L. Dias, Martina Mijuskovic, Sera Choi, Laura Kingham, Rachel A. Moore, Sarah M. Leiter, Rowena Guermech, Amanda Semerene, Aviva Grisby, Sophie Wool, Victoria Joslin, Arunthethy Mahendrayogam, Jonathan Abrams, Michael Gattens, Esther Blanco, Emmy L. Dickens, Charlotte Burns, Matthew J. Murray, John A. Tadross, Claire Trayers, Catherine E. Hook, Jennifer Becq, Taksina Newington, Ivana Armogida, Zoya Kingsbury, Pascal Grobecker, Ines Vitoriano, Mitchell A. Bekritsky, Jacqueline Weir, Louise J. Fraser, Mark Ross, David Bentley, Matthew Cullen, Sam Behjati, Jack Bartram, Sean J. Humphray, Patrick Tarpey, David Rowitch

**Affiliations:** 1 Cambridge University Hospitals NHS Foundation Trust, Cambridge, UK; 2 University of Cambridge, Department of Paediatrics, Cambridge, UK; 3 Illumina Inc., Cambridge, UK; 4 Medical Research Council Metabolic Diseases Unit, Institute of Metabolic Science-Metabolic Research Laboratories, University of Cambridge, Cambridge, UK; 5 Department of Histopathology, Cambridge University Hospitals NHS Foundation Trust, Cambridge, UK; 6 Illumina, Inc., San Diego, CA USA; 7 Wellcome-Sanger Institute, Hinxton, UK; 8 Great Ormond Street Hospital, London, UK; 9 The Hospital for Sick Children, Toronto, Canada; 10 Cedars-Sinai Guerin Children’s, Departments of Pediatrics and Neurosurgery, Los Angeles, USA

**Keywords:** Paediatric cancer, Cancer genomics, Personalized medicine

## Abstract

Whole-genome sequencing (WGS) has been shown to improve diagnosis beyond standard-of-care genetic tests and guide precision treatment for childhood cancer. While WGS is now offered for children with suspected cancer in England, clinical workflows typically return results after several weeks whereas clinical decisions are often required within days. Here we evaluate feasibility and efficacy of a rapid WGS (rWGS) research workflow with streamlined bioinformatics to deliver clinical genomic findings (NCT07201038, Active, not recruiting). In 54 children with suspected or confirmed malignancy, rWGS reduces mean end-to-end turnaround time from 42 to 3 days, whilst increase variant detection rate. Whereby rWGS detected 147 of 155 (95%) clinically actionable variants identified in the cohort, standard of care testing detected 138 of 155 (89%) variants. In a subset of 35 patients assessed prospectively, clinicians report that in 17 (49%), rWGS either (i) facilitates faster access to targeted therapy and risk stratification, or (ii) avoids unnecessary investigations and treatment escalation. These findings indicate feasibility and suggest clinical utility of rWGS when deployed as a first line diagnostic test in pediatric oncology.

## Abstract (web summary)

Whole-genome sequencing (WGS) can provide important benefits to childhood cancer patients, but clinical WGS workflows typically take weeks. Here, the authors evaluate the feasibility and efficacy of a rapid WGS research workflow with streamlined bioinformatics, reducing the mean turnaround time from 42 to 3 days without sacrificing the detection of clinically actionable variants.

## Introduction

The clinical utility of paired tumor and germline whole genome sequencing (WGS) of pediatric cancers has been demonstrated^1,2^ in childhood leukemia^1,3,4^ and solid cancers^3,5–8^. WGS has been shown to improve molecular diagnosis and management of pediatric cancers, enabling detection of prognostic and therapeutic variants beyond conventional molecular assays^3^. Rapid identification of prognostic or targetable drivers of pediatric cancer, including but not limited to *BCR-ABL*^9^, *MYOD1*^10^, *MYCN* amplification^11^, and *BRAF* alterations^12^ provides opportunities for precision treatment. For these reasons, the National Health Service (NHS) England Genomic Medicine Service (GMS) provides WGS diagnosis for all children with suspected cancer. However, the turnaround time (TAT) typically exceeds six weeks due to technical and logistical requirements, such as the need for fibroblast culture to obtain germline DNA for hematological malignancies and for DNA extraction and library preparation from fresh-frozen samples. Longer TAT limits the clinical utility of WGS because an imperative in pediatric oncology is to begin induction therapy within days of presentation. While several recent studies have indicated benefits of rapid genomics (e.g., same day sequencing) in pediatric critical care^13–15^, the feasibility of rapid WGS (rWGS) for children with suspected cancer has not been demonstrated.

Here, we show that rWGS can reduce TAT without sacrificing the detection of clinically actionable variants.

## Results

**Fig. 1:**
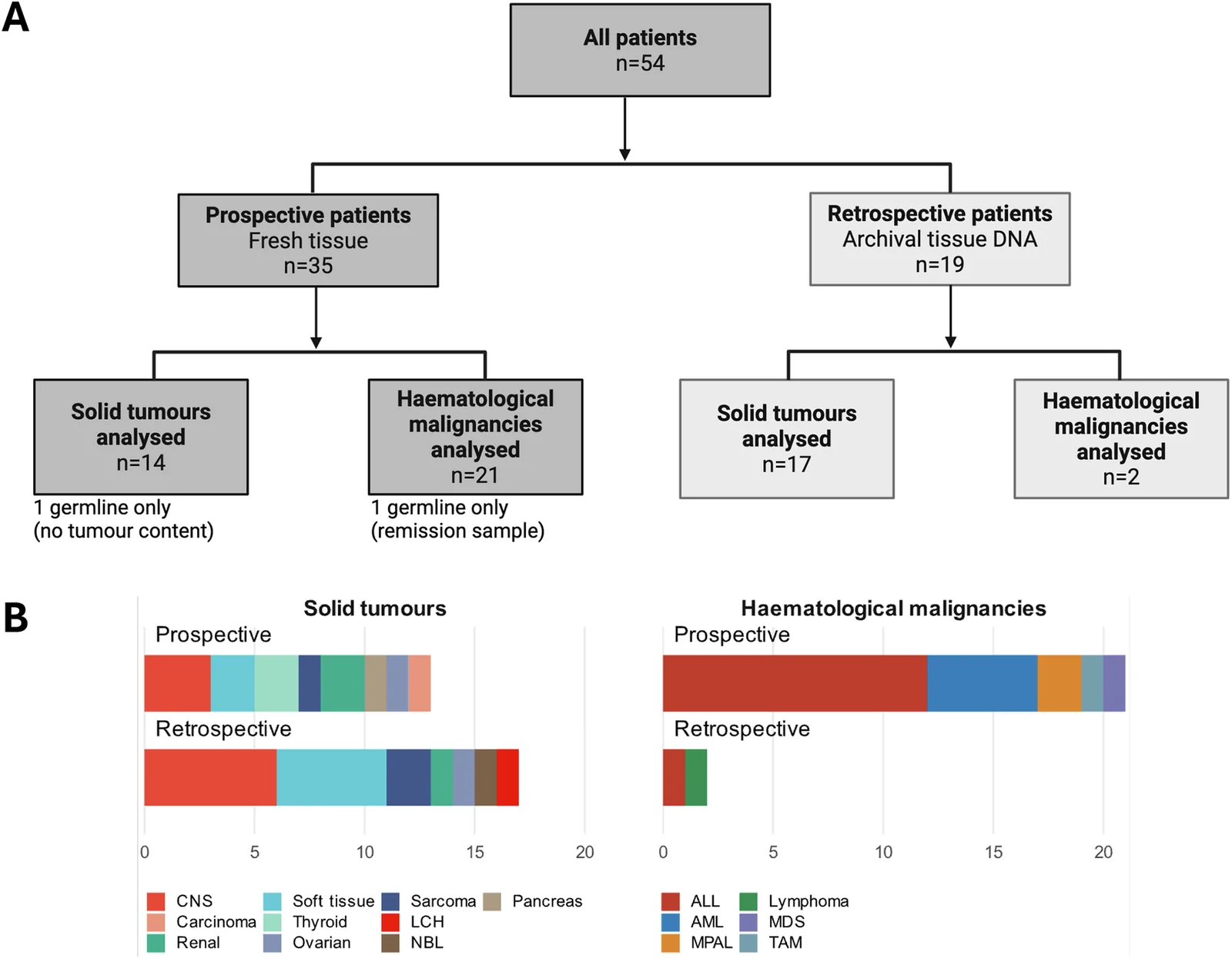
Study design, with 54 patients recruited overall. **A** Prospective patients were recruited at presentation and retrospective patients were selected for the availability of archival DNA and GMS-WGS results. **B** Bar plots showing a range of diagnostic groups represented in patients with solid tumors and hematological malignancies in prospectively and retrospectively recruited patients. Individual patient information is available in Supplementary Data 1. CNS central nervous system, LCH Langerhans cell histiocytosis, ALL acute lymphoblastic leukemia, AML acute myeloid leukemia, MPAL mixed phenotype acute leukemia, TAM transient abnormal myelopoiesis, MDS myelodysplastic syndrome.

Fifty-four children (mean age at diagnosis 6.9 years, range 0–17.5 years), with suspected or confirmed cancer were recruited between April 2023 and March 2025 (NCT07201038, Active, not recruiting); of these, 35 were prospectively enrolled with bone marrow, blood or fresh frozen tissue samples collected at the time of presentation or relapse, while 19 were enrolled using archival fresh frozen tissue (Fig. 1, Supplementary Data 1). All cases underwent rWGS deploying Illumina’s TruPath Genome (see Methods) on extracted DNA from solid tumors and blood for germline, or crude lysate from bone marrow (BM) and/or peripheral blood (PB) samples from hematological cancer patients. Median coverage depth was 137x for the tumor (inter-quartile range, IQR 51.2) and 84x for germline sample with rWGS, compared with 97x (IQR 17.4) and 42x, respectively, with GMS-WGS (Supplementary Data 1).

**Fig. 2:**
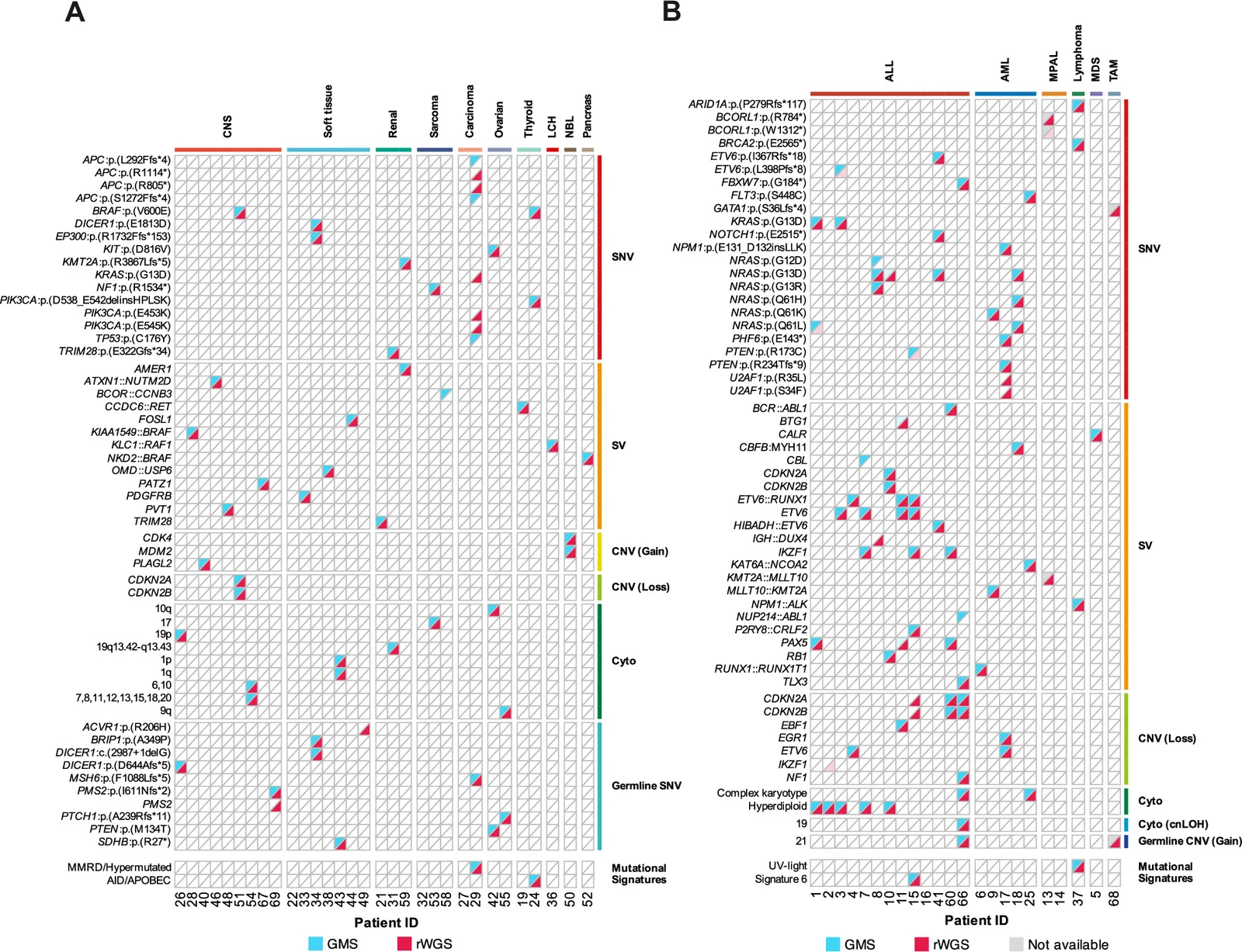
Clinically actionable variants detected by rWGS vs GMS-WGS. Oncoplots listing all clinically actionable somatic and germline variants. **A** Solid tumors (*n* = *31*); **B** hematological malignancies (*n* = 23). Variants with low variant allele frequency are shown: GMS (pale blue); rWGS (pink). Only germline variant comparison was performed for patient 69. Source data are provided as a Source Data file.

**Fig. 3:**
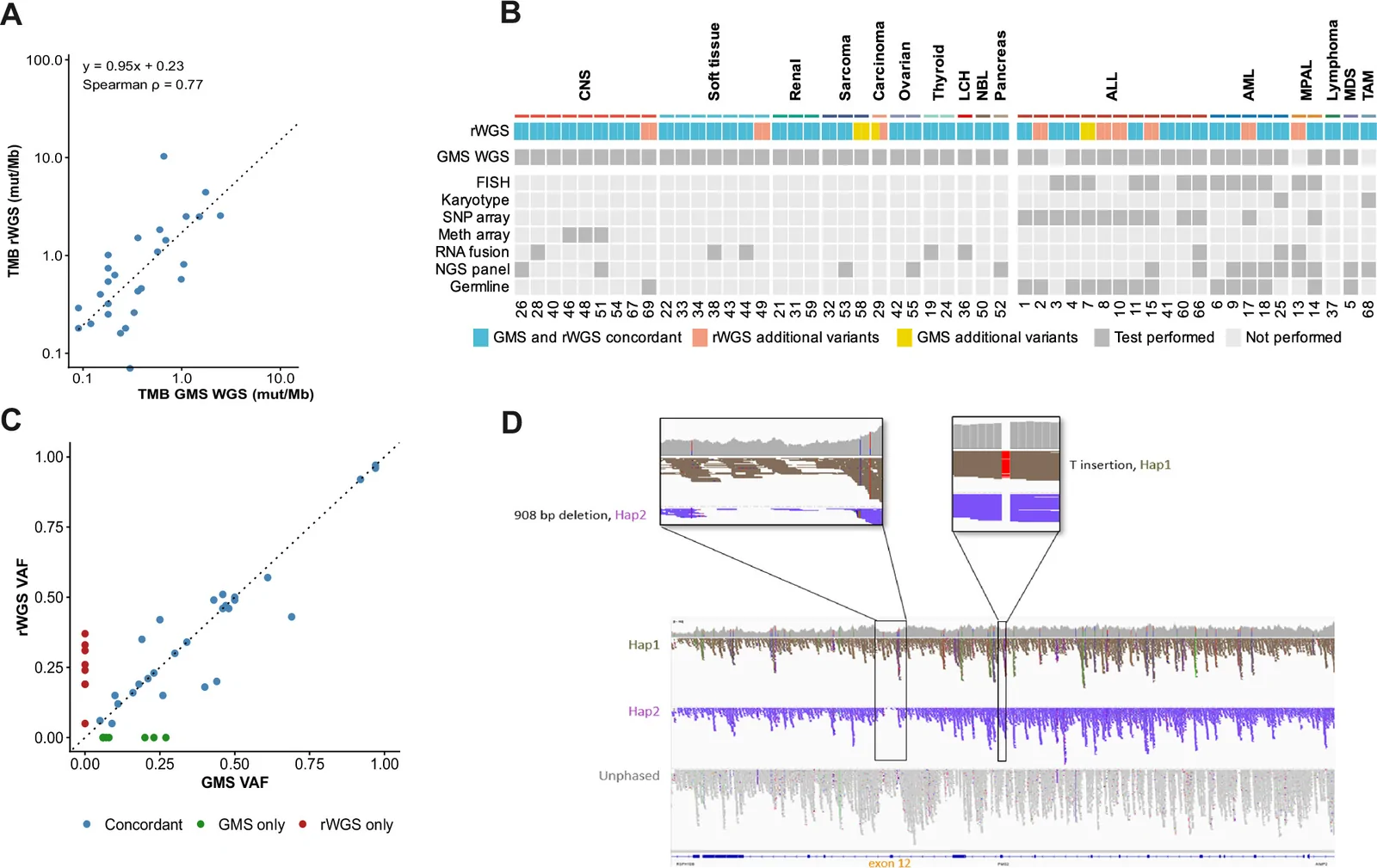
Technical comparison of rWGS with GMS-WGS and standard-of-care (SOC) assays. **A** Scatter plot representing tumor mutation burden for each patient as assessed by rWGS and GMS-WGS (*n* = 32 patients who had paired tumor-germline analysis). **B** Heatmap comparing clinically actionable variants yielded from rWGS compared with SOC assays performed, including GMS-WGS. **C** Scatter plot representing variant allele frequency for each clinically actionable variant. Only somatic single-nucleotide variants (SNVs) or insertions/deletions (Indels) are shown (*n* = 41 variants, 19 patients). **D** Identification and phasing of the germline *PMS2* variant in a patient with CMMRD (Patient 69). Phasing of both haplotypes in a difficult-to-capture region of the genome by rWGS. Source data are provided as a Source Data file.

Across 104 samples (Fig. 1, Supplementary Data 2), representing 37 distinct pediatric hematological and solid tumor entities (including leukemia, lymphoma, CNS tumors, neuroblastoma, Wilms tumor, and rarer entitiesm Fig. 1), rWGS captured 147/155 (95%) of clinically actionable somatic and germline variants found by any method, and recapitulated mutational signatures (Figs. 2A, 2B) and TMB estimation (Fig. 3A). The eight variants not detected by rWGS were clinically actionable (as defined by the NHS National Genomics Test Directory), and discordance was attributed to tumor heterogeneity or low variant allele frequency (Fig. 3C, Supplementary Table 1). In the clinical context, the absence of these variants did not alter the clinical diagnosis or management of each individual patient in the study, as there were sufficient supporting variants to define the diagnosis in each case. GMS WGS detected 138/155 (89%) variants (Fig. 2, Supplementary Data 2), thus there were 17 clinically relevant, reportable variants (as per Supplementary Data 4–6), which were only detected by rWGS. A complete variant-by-variant comparison of rWGS against NHS standard-of-care testing (including FISH, SNP array, targeted NGS, RNA fusion testing and GMS-WGS) is provided in Supplementary Data 2. Methylation analysis is not covered by WGS and when indicated (central nervous system tumors), was separately analysed.

We found that a major advantage was issuing a clinically actionable report as rWGS with shortened TAT, from a mean of 42 days (GMS-WGS for patients in this study, standard deviation, SD 30) to 3 days (SD 1.24); subgroup means were 63 days (hematological malignancies) and 29 days (solid tumors) for GMS-WGS, and 3 days for both subgroups for rWGS using the same definition (Supplementary Data 1, Supplementary Fig. 1). Time savings included removal of DNA extraction and fibroblast culture for leukemia patients and library synthesis for all patients, as rWGS allows direct application of DNA or, in this study, crude lysate onto the flowcell surface, providing both technical advantages and speed. rWGS showed equivalent detection across fresh and archival fresh-frozen specimens including low-cellularity tumors, which can be challenging to diagnose (Fig. 2, Supplementary Data 2).

All rWGS-specific findings were either confirmed by orthogonal assays before clinical implementation where clinically required, or verified by manual inspection of the corresponding GMS WGS raw data (Supplementary Data 2, column O). In the prospectively analysed cohort, 17/35 children (49%) had measurable improvements in care through reduced time to diagnosis, more accurate risk stratification, precision medicine, or avoidance of unnecessary procedures and treatments as recorded in routine multidisciplinary management discussions (Supplementary Table 2). Such improvements were judged a direct result of the rapid WGS data return or, in some cases, the technical advantages of TruPath Genome. Review of retrospective cases by three independent clinicians indicated potential benefit in 9/19 (47%) patients if these results had been available at the time of presentation (Supplementary Data 3). Some of the additional variants detected only by rWGS clinically benefited six patients (Fig. 3B, Supplementary Data 2), including Patient 13 (post-allogeneic stem cell transplant), who was precluded from GMS-WGS; rWGS diagnosed relapsed mixed phenotypic acute leukemia (MPAL), with subclonal lymphocytic variants not obvious on flow cytometry and conventional molecular testing.

Additional vignettes serve to illustrate the impact on clinical decision-making of rWGS. Two children (Patients 22 and 49) presenting with soft tissue lesions were diagnosed with non-malignant conditions, avoiding unnecessary and potentially harmful interventions. Both patients avoided invasive procedures, including insertion of central venous catheters for systemic treatment, which was deemed unnecessary once the rWGS findings were available. Patient 49 was found to harbor a pathognomonic *ACVR1* germline variant, confirming fibrodysplasia ossificans progressiva, where even minor trauma (e.g., surgical interventions, biopsy) can trigger disabling soft-tissue calcification. Another patient (Patient 52), with pancreatic acinic cell carcinoma, harbored a novel *NKD2-BRAF* fusion, enabling targeted MEK pathway inhibition. In the two children with Wilms tumor in our cohort, rWGS excluded germline predisposition in the absence of tumor samples, which are not routinely collected pre-chemotherapy as per European guidelines. This germline-only rWGS information at diagnosis could guide surgical planning, avoiding nephron-sparing strategies reserved for syndromic cases^16^. For a child with mediastinal lymphoma and life-threatening clinical presentation (Patient 66), molecular diagnosis from pleural fluid obviated a high-risk biopsy procedure.

rWGS significantly streamlined molecular diagnosis for leukemia patients. Tumor-only sequencing from BM or PB samples yielded minimal residual disease markers, cytogenetic subgroup risk stratification, and pharmacogenomic profiling within days, from a single sample. In an infant with Down syndrome and suspected transient abnormal myelopoiesis, rWGS confirmed somatic *GATA1* mutations and germline trisomy 21 on a PB sample alone, supporting surveillance and avoiding unnecessary chemotherapy, despite a rising blast count (Patient 68). In several cases, PB sequencing provided sufficient diagnostic information, potentially avoiding invasive BM biopsy (Supplementary Fig. 2).

Technical advantages of rWGS included germline variant phasing and improved detection within repetitive genomic regions. Long-range haplotype resolution assigned *PMS2* variants to distinct parental alleles in a child with constitutional mismatch repair deficiency (CMMRD), confirming the diagnosis and eligibility for immune checkpoint inhibition (Patient 69, Fig. 3D). A somatic-fusion filtering approach leveraging flowcell-proximity data confirmed novel fusions in difficult to map genomic regions such as *ATXN1::NUTM2D* (Patient 46, Supplementary Fig. 3), exemplifying potential for high accuracy detection of structural variants, pertinent for childhood cancer.

## Discussion

This study has evaluated the feasibility of rapid whole-genome sequencing to enhance clinical diagnostics in pediatric cancer. While there are no published UK guidelines on expected TAT for WGS in childhood cancer, TAT observed in the East GLH ranges from two-six-weeks, and the national average was four to six weeks from DNA or tumor sample being sent for sequencing to issuing of the clinical report (Supplementary Fig. 1). This study showed feasibility of issuing a report within 3 days via a simplified technical and informatics workflow. Data quality and reduced TAT enhanced the clinical diagnostic utility of WGS compared with our prior report^3^. While most samples analysed were paired somatic-germline, we found that another advantage of the rWGS pipeline was the ability to perform tumor-only or germline-first analysis. Clinician reports indicated that a 3-day WGS TAT improved (i) timely implementation of targeted therapy and pharmacogenomically guided treatment, (ii) earlier clinical trial enrollment, and (iii) avoidance of unnecessary procedures, and reported that (iv) 3-day TAT WGS would have benefited management in as many as half of the cases. Further studies are warranted to establish reproducibility, clinical benefit, cost-effectiveness, and scalability within national healthcare frameworks.

Our study was not powered to evaluate cost savings or designed to show clinical impact. Our study was limited by its relatively small size and single-center, non-randomized design. Therefore, a large-scale multi-center investigation is warranted.

## Methods

### Clinical study

This study included prospectively and retrospectively ascertained patients with cancer or suspected cancer aged <25 years in accordance with the NHSE guidelines for clinical-diagnostic WGS. We evaluated the feasibility and accuracy of an rWGS pipeline in a tertiary UK pediatric hematology-oncology center by benchmarking its performance in 54 cancer patient cases against NHS GMS WGS and other conventional molecular diagnostic assays, including FISH, SNP, and RNA sequencing.

The study was registered at ClinicalTrials.gov (NCT07201038, Active, not recruiting, https://clinicaltrials.gov/study/NCT07201038, registered 23rd Sep 2025), and approved by the Health Research Authority and Health and Care Research Wales (REC reference 22/WA/0336). Patients were recruited between April 2023 and March 2025. Written informed consent was obtained from all participants or their parents/legal guardians, including consent to the publication of patient-level age and sex information. Patients received no financial or other compensation for participation in the study. No sex/gender analysis formed part of this study as it was not relevant to the overall outcome.

Sample handling and DNA extraction for all solid tumor and archival samples was carried out in the NHS East Genomics Laboratory Hub, Cambridge University Hospitals NHS Foundation Trust (CUH), Cambridge, UK in accordance with the Human Tissue Act guidance. Tumor samples were obtained from diagnostic biopsies, and blood was obtained where germline analysis was required. Residual samples and/or DNA were returned to NHS GMS East Genomic Laboratory Hub (GLH) for long-term storage in accordance with Genomics England consent procedures and record of discussion (https://www.england.nhs.uk/wp-content/uploads/2021/09/nhs-genomic-medicine-service-record-of-discussion-form.pdf).

All rWGS data were generated using the Illumina TruPath^TM^ Genome technology (Cambridgeshire, UK). The platform enables rapid variant calling (structural variants (including fusions), single nucleotide variants, insertions/deletions, and copy number alterations), tumor mutational burden (TMB), and mutational signature analysis. Details of the sequencing technology and bioinformatic pipeline are provided below.

All rWGS data were analysed by clinical scientists at the NHS East GLH, with additional review by the Illumina laboratory. In parallel, samples underwent routine NHS GMS WGS and other conventional molecular diagnostics, including fluorescent in situ hybridization (FISH), single nucleotide polymorphism (SNP), RNA-sequencing, targeted next generation sequencing panels, real-time polymerase chain reaction (RT-PCR), and conventional immunohistochemistry. Clinically actionable variants identified by rWGS were orthogonally confirmed via NHS standard-of-care (SOC) molecular testing before the findings were used to inform patient management.

For the prospective cohort, samples from patients with hematological malignancies (except lymphoma) were sequenced directly from fresh peripheral blood (PB), bone marrow (BM), or, for Patient 66, pleural fluid crude lysate and analyzed in tumor-only mode. Retrospectively ascertained hematological malignancies and all solid tumors were sequenced using extracted DNA from fresh-frozen tissue and analyzed using paired tumor and germline samples (Supplementary Data 1). No formalin-fixed paraffin-embedded samples were used in this study.

TAT was defined in this study as the interval between sample availability of all samples required for WGS analysis - tumor and germline for paired analysis or tumor-only for hematology patients, and issuance of a clinically interpretable report. TAT included DNA extraction, sample transfer, sequencing and analysis for both methods. Only clinically actionable variants were reported as defined by the NHSE genomics test directory. All analyses and data visualization were performed using R Statistical Software (version 2024.12).

Data was integrated from different sources through a centralised data extraction person (A.V.), then validated by at least 3 team members (S.H., M.M., J.T., A.R.M., L.K., J.D.).

### rWGS Workflow

#### DNA extraction and crude lysate preparation

Genomic DNA from the PB and/or BM was extracted using the QIAmp Blood DNA Mini kit and MagAttract® HMW DNA kit (Qiagen), following the manufacturer’s protocols. A total of 200 µl of PB and 100 µl of BM (diluted with 100 µl of PBS) were used as input for the extractions. Genomic DNA from frozen tumor samples was extracted using the MagAttract® HMW DNA kit (Qiagen, Germany), following the manufacturer’s protocols, with a lysis incubation at 56 °C for 2 hours. Depending on tissue availability, between 5 mg and 20 mg of tissue were used as input for the extractions. DNA concentration was determined using the Qubit dsDNA BR Assay kit (ThermoFisher Scientific), and quality was assessed using the genomic DNA ScreenTape assay (Agilent Technologies). TruPath Genome is optimized to 350 ng DNA per lane, which is sufficient for germline sequencing depth. In this study, 2 lanes per tumor sample were combined, resulting in high-quality, high-depth somatic sequencing results.

PB and BM samples in the prospective cohort were sequenced directly from crude lysates in this study using 25 or 15 µl of each sample, respectively, and mixed with the lysis buffer containing proteinase K.

#### TruPath Genome sequencing

Illumina WGS mapped read technology (TruPath Genome)^17^ utilizes on-flow cell library preparation and uses proximity information from neighboring nanowells to generate long-range genomic data using standard sequencing by synthesis (SBS). Separate library preparation is eliminated by the use of flow cell-bound transposomes, which capture and tag long molecules of DNA as they are flowed onto the flow cell surface, and ensure that adjacent regions in a sample’s genome remain physically proximal on the flow cell.

Clustering and SBS were performed as per standard, resulting in high-quality polymerase chain reaction (PCR) free short-read sequencing data. No modifications to the instrument hardware were required^17,18^.

The resulting reads from neighboring clusters can be reconstructed into an interspersed version of the original DNA template molecule, enabling haplotype phasing, improved read mapping and higher structural variant detection accuracy. The technology is fully supported for germline genomes and applied in this study to tumor genome data using research workflows to demonstrate the feasibility of the approach.

Mean sequencing depth was 148x for somatic and 83x for germline.

#### Alignment and variant calling

Whole-genome sequencing reads were aligned to the linear version of the GRCh38 reference genome. Hematological cancer samples were analyzed in the tumor-only mode using DRAGEN somatic pipeline version 4.0, with likely germline small variants called based on their population frequency and gnomAD count > 50^19^. Solid cancer samples were analyzed in the paired tumor/normal mode using DRAGEN somatic pipeline version 4.2^20,21^ and DRAGEN germline pipeline version 4.2 to call germline variants^22^. For all sample types, copy-number variant (CNV) calling was done in the ‘heterogeneous’ mode, allowing subclonal CNV calls. Full chromosome and chromosome arm copy number changes were assessed visually from coverage and B-allele ratio plots. Systematic noise files were used to reduce false-positive small and structural variant calls. Only variants with VCF PASS status were included in the downstream analyses and reporting.

#### Tumor content estimation

Tumor purity was initially estimated by the DRAGEN pipeline, fitting the purity/ploidy model using B-allele frequencies and coverage data across the whole genome. It was then manually re-estimated using genome-wide plots of B-allele frequencies, coverage levels, and somatic small variant allele frequency (VAF) distributions. When the two estimates disagreed, as sometimes observed in heterogeneous samples, manual estimation was preferred. In cases where DRAGEN pipeline couldn’t provide a confident purity estimate, manual estimate was used as above or by utilizing VAF of the known driver mutation.

#### Annotation and variant filtering

Germline and somatic variants were annotated by Nirvana version 3.9.0^23^ using the Ensembl 91 transcript reference database. Pertinent variants (excluding pharmacogenomic variants) were triaged via consequence annotations (SO terms): feature elongation, non-synonymous coding sequence variant, unidirectional gene fusion, bidirectional gene fusion, transcript ablation, canonical splice acceptor/donor variants, stop gained, transcript truncation, frameshift variant, stop lost, start lost, transcript amplification, in-frame insertion, in-frame deletion, missense variant, protein altering variant, splice region variant, incomplete terminal codon variant, copy number increase, copy number decrease, copy number change, transcript variant. Somatic and germline structural variants, other than inter-chromosomal translocations, were considered only if they would disrupt the exons of one of the genes or were located within its promoter region.

Pertinent germline and somatic variants were triaged further using the gene and genomic region of interest list provided for each cancer and/or variant type (Supplementary Data 4-6 for somatic variants in hematological cancers, somatic variants in solid cancers and germline variants, respectively). If no findings were identified, the list was extended to COSMIC cancer gene census v83 (https://www.sanger.ac.uk/data/cancer-gene-census/). In line with standard GMS WGS variant reporting, only the variants listed in Supplementary Data 4-6 were reported, to comply with the consent process for standard WGS (https://www.england.nhs.uk/wp-content/uploads/2021/09/nhs-genomic-medicine-service-record-of-discussion-form.pdf) and this study.

#### Calculation of tumor mutational burden (TMB) and mutational signatures

TMB and mutational signatures have been analysed only in cases with both tumor and germline sample available, excluding hematological malignancies where only tumor-only analysis has been possible due to the lack of germline material.

TMB was calculated as the sum of somatic SNVs and indels, divided by the GRCh38 genome size in megabases. Variants with a population frequency >1% in the 100, 000 Genomes Project were excluded on the basis that they likely represent germline variants.

Mutational signature analysis was performed by calculating the fraction of each somatic mutation type in a specific class and then applying non-negative least squares to decompose the fraction data from each sample into the operative mutational signatures. To calculate fractions, single base substitutions were divided into 96 classes, double base substitutions into 78 classes, and indels into 83 classes using SigProfilerMatrixGenerator^24,25^. Reference mutational signatures used for decomposition were from the COSMIC 3.3 release^26^. Only signatures supported by >1000 single-base substitutions or >200 indels were considered significant.

#### Tertiary analysis of variants

Data files with annotated variant calls were returned to scientists via an interactive HTML file developed by Illumina. Variant calls were intersected with pre-defined lists of clinically relevant genes (Supplementary Data 4–6) to overlay pertinent annotation and direct focus based on the clinical diagnosis.

Though the focus of the analysis was aimed at known cancer genes, particularly those considered “on target” for the referred cancer type, other variants outside annotated cancer genes were considered if they presented as compelling. This may include novel translocations which present as conspicuous in otherwise quiescent genomes. In this way, the analysis was agnostic of the suspected diagnosis as no variants are excluded from consideration. Visual inspection of fluid genome plots was employed to confirm the validity of copy-number and structural variation when necessary. Inspection of SNV was only performed if the supporting data is equivocal.

Completed analysis was typically rapid and presented for discussion at a weekly pediatric GTAB prior to issuing a formal report

#### DUX4r calling

*DUX4* rearrangements were called using the custom caller Pelops (available in DRAGEN version 4.4 + ). SRPB threshold of 10 was used to call samples positive for *DUX4-IGH*, while a threshold of 15 was used for *DUX4*-other. False-positive regions were filtered using a blacklist as described in Grobecker et al.^27^.

### Analysis 1: Genetic variant detection by GMS and rWGS platforms

This analysis was performed using two independent Tables in an Excel File (Solid and Hematological cancer types) contains patient data, genetic variants, diagnostic subgroups, and detection across GMS and rWGS platforms.

- **Environment Setup**: The heatmaps were generated in R script using libraries *readxl*, *stringr*, *rvest*, *ggplot2*, *ComplexHeatmap*, *reshape2*, *dplyr*, *grDevices*, and *circlize*.
- **Data Loading and Preprocessing**: The dataset was loaded from an Excel file. Data was aggregated to create unique entries for each genetic variant per patient. A custom function converted protein change notations in the *Variant* column from three-letter to one-letter amino acid codes. “Yes”/“No” values in the GMS and rWGS columns were converted to numeric 1/0.
- **Heatmap Visualization**: Two data matrices were created to represent the presence or absence of each variant for each patient on GMS and rWGS platforms, respectively. A primary heatmap was built using ComplexHeatmap with a custom cell function to render each cell as a rectangle split by a diagonal line. The upper triangle was colored based on the GMS value, and the lower triangle by the rWGS value. A second heatmap was created to display the presence of mutational signatures (MMRD/Hypermutated, AID/APOBEC, UV-light, Signature 6). The two heatmaps were combined vertically and the final figure was exported as an SVG file.

### Analysis 2: Comparative analysis of WGS and other assays

This analysis was performed using an Excel File contains patient data, diagnostic subgroups, and detection across different genomic assays.

- **Environment Setup**: The analysis was conducted in R using the *readxl*, *dplyr*, *ComplexHeatmap*, *circlize*, and *grid* packages.
- **Data Structuring for Visualization**: Data was loaded and converted to a numerical matrix, with patient IDs as row names, the assays detection as columns and missing values imputed to 0. The matrix was transposed so that assays detection were rows and patients were columns. The data was then partitioned into three matrices (Ultrafast WGS data, Routine NHS WGS and all other assays). The top two heatmaps display the detection in Ultrafast WGS and Routine NHS WGS, respectively. The larger heatmap displays all other assays performed in GMS, with rows grouped by assay type. All heatmaps share the same column order, which is determined by the Diagnosis Subgroup and are annotated accordingly. The final figure is a composite of three vertically concatenated heatmaps and was saved as a high-resolution SVG file.

### Analysis 3: Turnaround time (TAT) analysis

This analysis was performed using an Excel file containing turnaround time (TAT) data for Solid and Hematological cancer types and for GMS WGS and rWGS platforms.

- **Software and Libraries**: The analysis used R with the *readxl*, *dplyr*, and *ggplot2* packages.
- **Data Processing and Visualization**: Data was imported and grouped by Sample Type (Solid and Hem), Platform, and sample processing stage. Summary statistics, including the number of observations, mean TAT, and standard deviation, were calculated for each Sample Type. A bar chart was generated using ggplot2 to represent the mean TAT, with error bars indicating the 95% confidence intervals. To visually separate each sample processing stage, the mean values for the “Samples > Sendout” stage were negated on the plot. The final plot was faceted by Sample Type and saved as an SVG file.

### Analysis 4: Variant allele frequency (VAF) analysis

This analysis was performed using an Excel file containing patient data, diagnostic subgroups, and genetic variant data – including variant type and variant allele frequency (VAF) – for both Solid and Hematological cancer types across the GMS WGS and rWGS platforms.

- Software and Libraries: The scatter plot was generated using R script with the following libraries: *readxl*, *dplyr*, and *ggplot2*.
- Data Processing and Visualization: The dataset was imported and filtered to include only somatic single-nucleotide variants (SNVs) and insertions/deletions (Indels), based on the “Origin” and “Variant type” columns for both solid and hematological cancer types. VAF values were converted to a numeric format and visualized as a scatter plot using ggplot2, with points colored by concordance between GMS WGS and rWGS platforms.

### Quantification and statistical analysis

#### Heatmap-based visual analysis (analyses 1 & 2)

These analyses focused primarily on data aggregation and visualization rather than formal statistical testing. The core of the method involved transforming data from a long format into wide-format matrices suitable for heatmaps. This was achieved using functions like dcast or by transposing the data frame. The resulting matrices contained values indicating the detection of a variant in an assay or platform (1 for presence, 0 for absence in Analysis 1, and 1 for rWGS and GMS concordance, 0.5 for rWGS additional variants, and 0 for GMS additional variants in Analyses 2).

No statistical clustering was performed. Instead, the rows and columns of the heatmaps were explicitly ordered based on predefined factors such as Variant type and Diagnosis Subgroup to facilitate qualitative comparisons between groups and platforms.

### Statistical analysis of turnaround times (analysis 3)

For the TAT analysis, 95% confidence intervals (CI) for the mean TAT were calculated for each group using the t-distribution. The formula used was:

1 $${{\mbox{CI}}}={{\mbox{mean}}}\_{{\mbox{TAT}}}\pm {{\mbox{t}}}_{0.975{{\mbox{,n-}}}1}\times \frac{{{\mbox{sd}}}\_{{\mbox{TAT}}}}{\sqrt{{\mbox{n}}}}$$

where t_0.975,n−1_ is the critical value from the t-distribution for a 95% confidence level with n − 1 degrees of freedom. The calculated means and confidence intervals were then visualized as bar plots with error bars.

### Reporting summary

Further information on research design is available in the Nature Portfolio Reporting Summary linked to this article.

## Supplementary information

Supplementary Information NEW

Supplementary Data NEW

Reporting Summary

Transparent Peer Review file

## Source data

Source Data for all Figures

## Data Availability

The de-identified patient-level information and summary metrics used for analysis in this study are available in Supplementary Data 1. Variant data used for signature analysis are provided in Supplementary Data 2. Source data are provided as a Source Data file, and available in the Apollo Database via DOI 30246127 https://doi.org/10.17863/CAM.126857.2. Data dictionaries for Figs. 2 and 3, and Supplementary Figs. 1 and 2 are available in Supplementary Data 7−12. The raw sequencing data are available under restricted access. Requests for raw sequencing data, variant calls, Genomics England (GEL) signature analysis, quality metrics and a summary of findings submitted to Genomics Laboratory Hubs can be made via the Genomics England Research Environment, a secure cloud workspace. To access this workspace, researchers must apply for membership of the Genomics England Research Network via an academic institution as per the following steps: first, a signed participation agreement must be submitted by the institution to gecip-help@genomicsengland.co.uk. Then, following selection of an appropriate research domain, an online application should be submitted. Applications will be reviewed within ten working days, following which institutions must validate the researcher’s affiliation. If approved, access to the Genomics England Research Environment will be granted following successful completion of an online Information Governance Training module. Source data are provided with this paper.

## Code availability

R scripts used for analysis and figure generation, and all relevant source data generated in this study have been deposited in the **Apollo database**, https://doi.org/10.17863/CAM.126857.2, under the MIT license. Source data are provided with this paper.
