# Supplementary material for: Rapid Whole Genome Sequencing Enhances Pediatric Cancer Diagnosis
Source: Nat Commun. 2026 Oct 6;17:10078. doi: 10.1038/s41467-026-77918-x (PMC13642919; doi:10.1038/s41467-026-77918-x)
Supplement: Supplementary file 1 [file 41467_2026_77918_MOESM1_ESM.pdf]

Supplementary Note 1

Detailed Description of the code

Figure 1A: Solid Tumour Genomic Comparison

- **Purpose**: This script creates a complex heatmap to compare the detection of genomic variants in solid tumours across two platforms: GMS WGS (Genome Medicine Sweden) and rWGS (rapid WGS).
- **Data Processing**:
  - Aggregates variant data by Patient ID (PID) and Diagnosis Subgroup.
  - Converts 3-letter amino acid codes to 1-letter codes (e.g., "Ala" to "A") for concise labelling.
  - Converts platform detection status ("Yes", "No", "NA") into numeric values for heatmap generation.
- **Visualization**: Generates a heatmap where each cell is split into two triangles—one representing GMS (cyan) and the other rWGS (red)—to visually highlight concordance and discrepancies in variant detection. It also includes top annotations for diagnosis and variant types.

Figure 1B: Haematological Genomic Comparison

- **Purpose**: Similar to Figure 1A, this script focuses on haematological malignancies (like ALL and AML) and includes additional mutational signature analysis.
- **Data Processing**:
  - Processes detection statuses including "Low VAF" and "-" (not available).
  - Handles mutational signatures such as UV-light and Signature 6 (associated with MMRD).
- **Visualization**: Uses a split-cell heatmap to compare platform performance specifically for blood cancers. It includes a specialized legend for platforms and signatures, with distinctive colours for different diagnosis subgroups.

Figure 2A: TMB Correlation Analysis

- **Purpose**: Analyses the correlation of Tumour Mutational Burden (TMB) between the GMS and rWGS platforms, after removing the two upper rightmost points.
- **Analysis**:
  - Performs linear regression (lm) to calculate the slope, intercept, and Spearman ρ value.
  - Supports different scale modes: linear, log10, or pseudo_log.

- **Visualization**: Produces a scatter plot with a regression line. The plot includes statistical annotations (the equation and R^2) to quantify the strength of the relationship between the two sequencing methods.

Figure 2B: Platform Concordance Heatmap

- **Purpose**: Visualizes the high-level concordance between platforms across all tested patients.
- **Data Processing**: Uses categorical codes to represent different levels of agreement: concordant, additional variants in rWGS, or additional variants in GMS.
- **Visualization**: Creates a highly customized heatmap with coloured blocks representing the "concordance status" for various diagnostic categories. It uses a specific palette where cyan represents concordance and orange/yellow represent platform-specific additional findings.

Figure 2C: Variant Allele Frequency (VAF) Comparison

- **Purpose**: Compares quantitative values (such as VAF) between two platforms or samples.
- **Visualization**: A scatter plot that includes an identity line (a dotted line where x = y). Points are color-coded by a grouping variable (e.g., diagnosis or variant type) to show how closely the values from both platforms align.

Figure S1: Turnaround Time (TAT) Analysis

- **Purpose:** Measures the efficiency of the clinical pipeline from sample receipt to data delivery.
- **Data Processing:**
  - Calculates the time spent in two main phases: "Samples > Sendout" and "Sendout > Data".
  - Computes confidence intervals (CI) for the TAT means.
- **Visualization:** Generates a stacked bar chart faceted by sequencing type. It uses error bars to display the standard deviation (SD) for the total turnaround time.

Figure S2: Bone Marrow (BM) vs. Peripheral Blood (PB)

- **Purpose**: Evaluates whether variants detected in bone marrow are also found in peripheral blood, specifically for leukaemia.
- **Data Processing**:
  - Aggregates blast percentages and tumour purity for both BM and PB samples.

  - Uses binary flags (1 for detected, 0 for not detected) for the comparison.
- **Visualization**: Combines a heatmap showing variant detection (split by BM and PB) with top bar plot annotations that display the blast counts/percentages for each patient.

Supplementary Tables

Patient ID	Diagnosis	Discrepant variant	VAF	Detected by	Reason for discrepancy
29	Colorectal carcinoma	APC: c.2413C>T; p.(Arg805Ter)	0.37	rWGS	Intratumoral heterogeneity, different slices sequenced by each platform
		APC: c.3340C>T; p.(Arg1114Ter)	0.33		
		PIK3CA: c.1357G>A; p.(Glu453Lys)	0.24		
		PIK3CA: c.1633G>A; p.(Glu545Lys)	0.31		
		KRAS: c.38G>A; p.(Gly13Asp)	0.26		
29	Colorectal carcinoma	APC: c.875dupT; p.Leu292PhefsTer4	0.2	GMS	
		APC: c.3814dupT; p.Ser1272PhefsTer4	0.23		
		TP53: c.527G>A; p.Cys176Tyr	0.27		
1	B-ALL	NRAS: c.182A>T;p.Gln61Leu	7.00E-02	GMS	Low VAF, different pulls from bone marrow sample
		ETV6: c.1193_1195delinsCGGAG; p.Leu398ProfsTer8	0.06	GMS	
3	B-ALL	NRAS: c.35G>A;p.Gly12Asp	0.06	GMS	
8	B-ALL	NRAS: c.38G>A;p.(Gly13Asp)	0.05	rWGS	
10	B-ALL	PTEN: c.517C>T;p.Arg173Cys	0.08	GMS	
15	B-ALL (relapsed)	U2AF1: c.104G>T;p.Arg35Leu	0.19	rWGS	
17	AML	U2AF1: c.101C>T;p.Ser34Phe	0.19	rWGS	

Supplementary Table 1: List of the 15 variants from 7 patients that were discordantly identified between rWGS and GMS-WGS. B-ALL, B-cell acute lymphoblastic leukaemia; AML, acute myeloid leukaemia; VAF, variant allele frequency.

Patient ID	Clinical presentation	rWGS benefit	rWGS variant	rWGS-guided potential for impact on treatment
**Accelerated Diagnosis**				
13	Mixed Phenotypic Acute Leukaemia	WGS post allogeneic HSCT, not possible with GMS WGS	*KMT2A-MLLT10*<br>*BCOR-1*	MPAL-directed treatment rather than lymphoid alone; flow cytometry did not detect small myeloid clone
59	Renal tumour, relapsed	Rapid diagnosis	Mixed molecular features of RCC and Wilms: *AMER1, KMT2A*	Continuation of Wilms directed treatment
66	Mediastinal mass, patient in extremis,	Rapid diagnosis; *rWGS from pleural fluid - flow cytometry not feasible on limited tumor cells*	NUP214-ABL1 fusion<br>*TLX3*-rearrangement<br>*CDKN2A/CDKN2B* loss<br>Complex karyotype	Confirmation of T-cell lymphoma, avoiding biopsy
68	Neonate with myeloid blasts	Rapid diagnosis	*GATA1* somatic and trisomy21 germline mutations	Confirming TAM, excluding AML
**Prognosis**				
2	B-ALL	Additional variant	*IKZF1*	*Stratified as high risk*
**Treatment De-escalation/Avoidance**				
22	Soft tissue lesions	Rapid diagnosis	Normal genome (somatic and germline), not obvious on histopathology alone	Observation only – fat necrosis
49	Soft tissue lesion, interscapular	Rapid diagnosis; Additional variant	Germline *ACVR1* variant Detected prior to biopsy histology being available	Fibrodysplasia Ossificans Progressiva, avoid surgery, central line insertion and chemotherapy, both potentially mutilating
8	B-ALL	Additional variant	*IGH-Dux4* rearrangement, expected delayed but good response	Non-escalation of treatment with positive MRD at end of induction, prior to GMS-WGS availability
**Targeted Treatment**				
24	Papillary thyroid carcinoma	Rapid treatment target detection	*BRAF V600E* and *PIK3CA* variants	BRAF inhibition in incompletely resected tumour
28	Pilocytic astrocytoma	Rapid treatment target detection	*KIAA1549-BRAF* fusion	Eligible for targeted therapy trial (Tovorafenib, Firefly2)
29	Colorectal carcinoma	Rapid diagnosis; Additional variants	*PIK3CA* somatic variant; confirmation of CMMRD (germline *MHS6* variant)	Eligible for PIK3CA inhibitor<br>Avoidance of ionising radiation
52	Pancreatic acinic cell carcinoma	Rapid treatment target detection	*NKD2-BRAF* fusion (novel partner)	Eligible for MEK inhibitor in context of known poor prognosis
60	B-ALL	Rapid treatment target detection	*BCR-ABL* fusion, detected prior to FISH	Early Tyrosine kinase inhibition, on day 1 of treatment
**Cancer Risk - germline variant detection**				
14	Mixed Phenotypic Acute Leukaemia	Germline only analysis	No germline bone marrow failure variants	Standard HSCT conditioning rather than reduced-intensity
21	Renal tumour, family history suggestive of cancer predisposition	Germline only analysis	Radiologically consistent with Wilms Tumour, no germline predisposition	No biopsy, non-nephron sparing nephrectomy post neo-adjuvant chemotherapy, prior to tumor histology
27	Nasopharyngeal carcinoma	Germline only analysis	No DPYD germline variant	Pharmacogenomically guided 5-fluorouracil dosing
26	Pineoblastoma	Rapid diagnosis - DICER1 syndrome	Germline *DICER1* variant	Would avoid ionising radiation from diagnosis where feasible

Supplementary Table 2: Clinical impact of rWGS. Prospectively sequenced patients with a potential change in management guided by rWGS. Highlighted patients represent those with additional variants detected only by rWGS, and non-highlighted patients benefited from the rapid return of WGS data. B-ALL, B-cell acute lymphoblastic leukaemia; MRD, minimal residual disease; RCC, renal cell carcinoma; TAM, transient abnormal myelopoiesis; AML, acute myeloid leukaemia; MPAL, mixed phenotype acute leukaemia; HSCT, hematopoietic stem cell transplant.

Key Resources Table

**RESOURCE**	**SOURCE**	**IDENTIFIER**
**Software and Algorithms**		
R	R-Core Team	v4.5.0
RStudio	Posit	v2025.05.0+496
readxl	CRAN	v1.4.5
dplyr	CRAN	v1.1.4
ggplot2	CRAN	v3.5.2
ComplexHeatmap	Bioconductor	v2.24.0
circlize	CRAN	v0.4.16
stringr	CRAN	v1.5.1
rvest	CRAN	v1.0.4
reshape2	CRAN	v1.4.4
grDevices	R-Core Package	v4.5.0
grid	R-Core Package	v4.5.0
Analysis Scripts	This study	See **METHOD DETAILS**
**DNA extraction**		
QIAmp Blood DNA Mini kit	Qiagen	
MagAttract® HMW DNA kit	Qiagen	
Qubit dsDNA BR Assay kit	Thermo Fisher Scientific	
DNA ScreenTape assay	Agilent Technologies	
**Sequencing data alignment and variant calling**		
DRAGEN somatic pipeline version 4.0		
DRAGEN somatic pipeline version 4.2		
DRAGEN germline pipeline 4.2		
Nirvana version 3.9.0		

Supplementary Table 3: Key Resources Table

Supplementary Figures

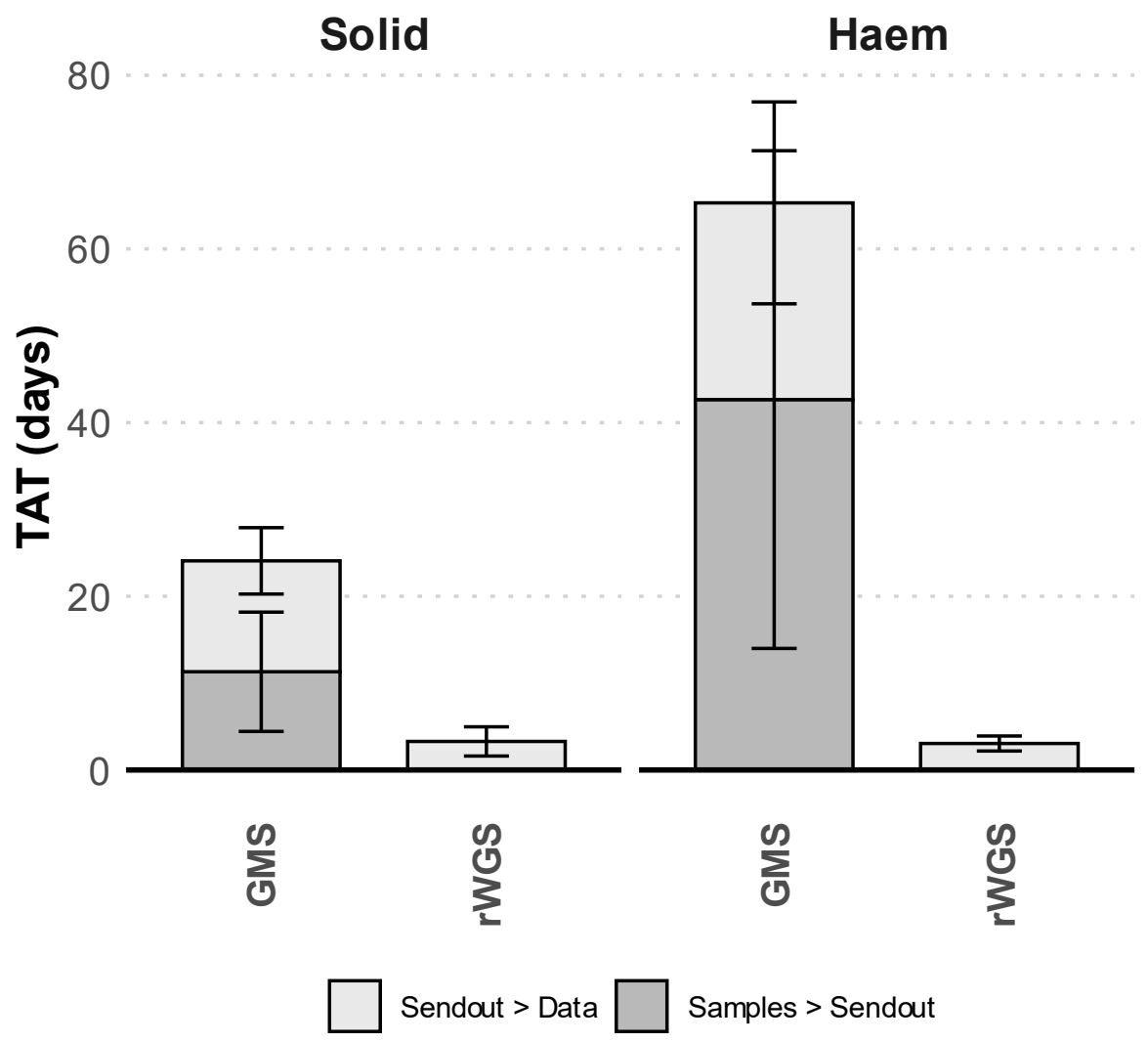

Supplementary Figure 1: Bar plots representing mean time (days) from paired tumor and germline sample availability (or tumor only for hematological malignancies in rWGS) to sequencing sendout (dark grey bars) to issuing of a clinically actionable report (light grey bars). Error bars represent standard deviation (SD). Solid tumours: GMS n=26, rWGS n =14 ; Haematological malignancies: GMS n=20, rWGS n=21. Source data are provided as a Source Data file.

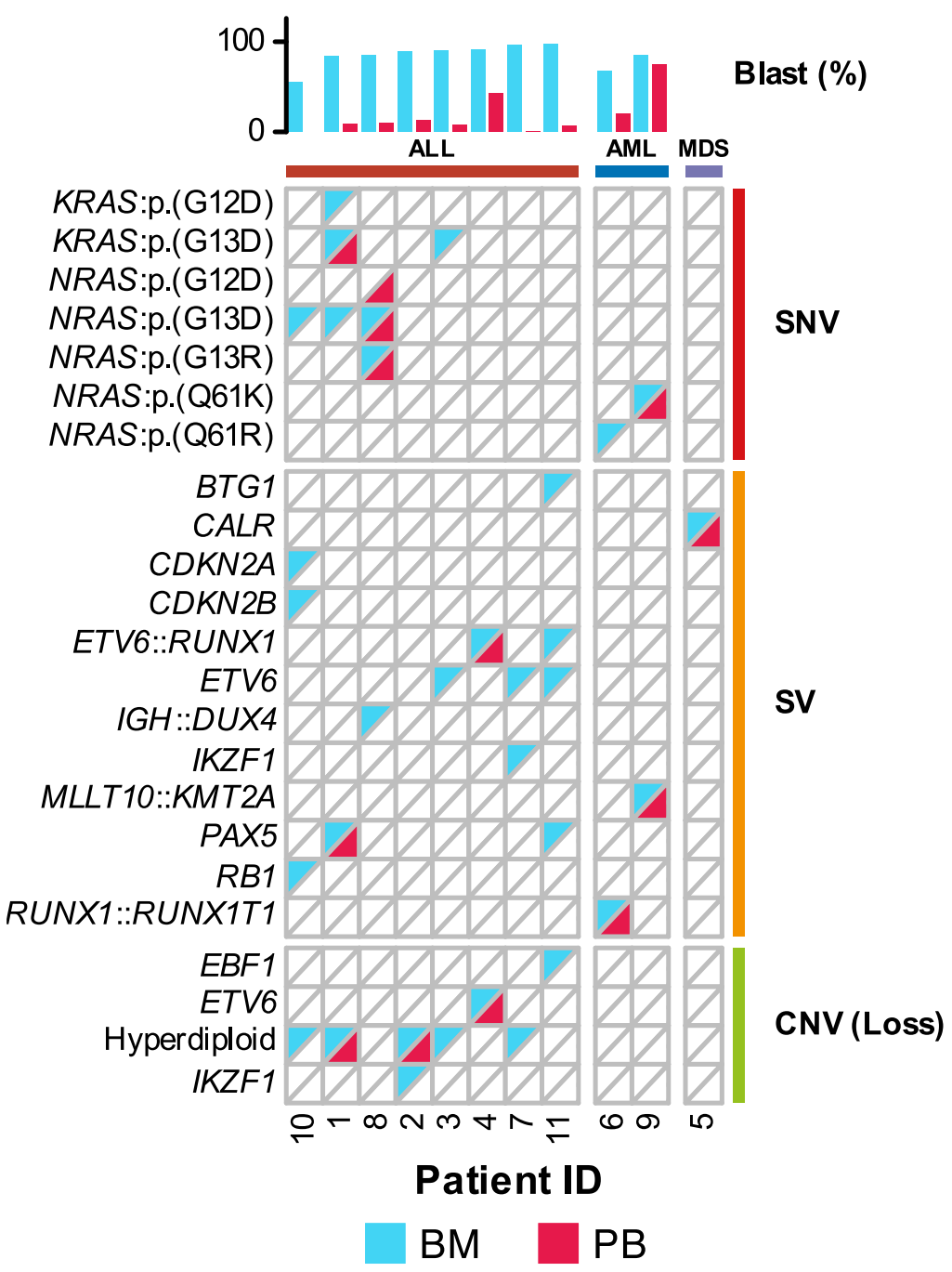

Supplementary Figure 2: Oncoplot showing clinically actionable variants (left) grouped by variant type (right) detected by rWGS in the bone marrow (BM) and peripheral blood (PB) from n=11 children with hematological malignancies. Blast percentage is shown in the top bar and is calculated based on flow cytometry of BM samples. Source data are provided as a Source Data file.

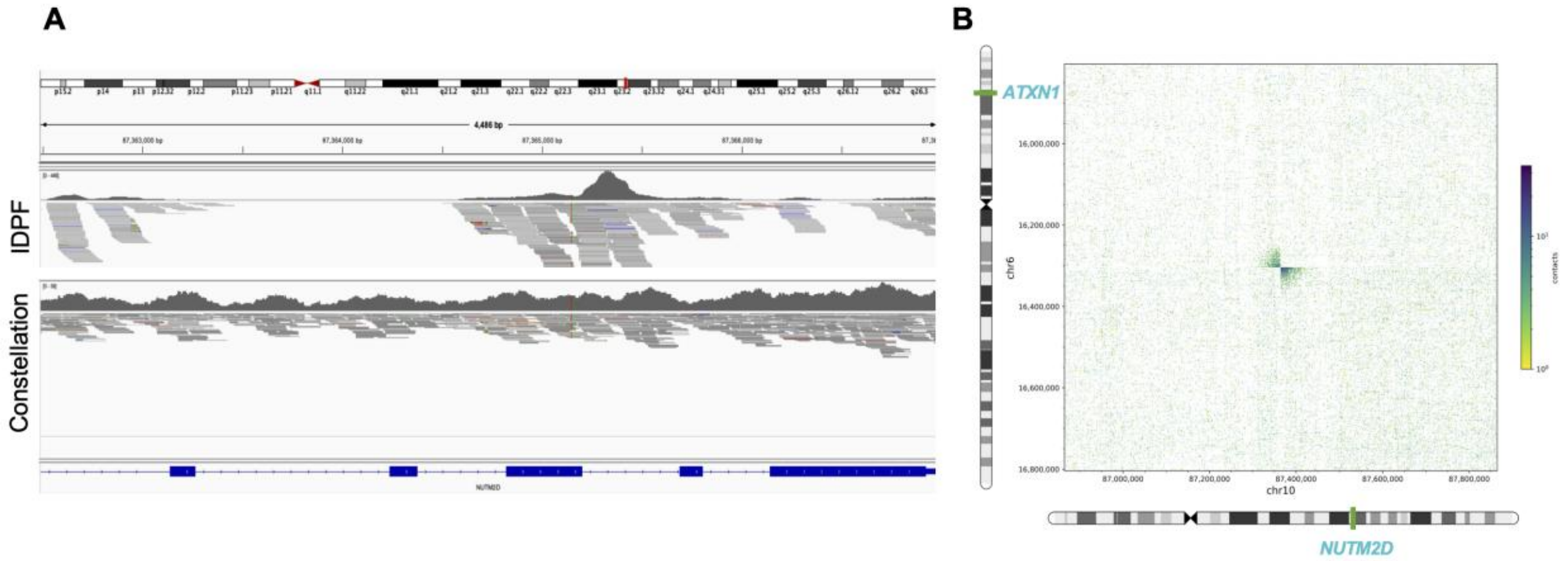

Supplementary Figure 3: Additional insights specific to novel Illumina TruPath technology.
(A) Using a somatic proximity pipeline, TruPath closes coverage gaps in high homology *NUTM2D* gene. Reads with mapping quality >20 are shown. IDPF, Illumina DNA PCR-free library preparation protocol (standard). (B) Reciprocal *ATXN1::NUTM2D* fusion in Patient 46 called with DRAGEN pipeline is confirmed with long-range interactions obtained from flowcell-proximity data. Fused regions in both derivative chromosomes are represented as darker-shaded areas of the plot. Breakpoint positions: chr6:16303813-chr10:87364730 (*ATXN1::NUTM2*), chr10:87362944-chr6:16303450 (*NUTM2::ATXN1*).
