# Supplementary material for: Rapid Whole Genome Sequencing Enhances Pediatric Cancer Diagnosis
Source: Nat Commun. 2026 Oct 6;17:10078. doi: 10.1038/s41467-026-77918-x (PMC13642919; doi:10.1038/s41467-026-77918-x)
Supplement: Supplementary file 3 [file 41467_2026_77918_MOESM4_ESM.pdf]

nature portfolio

Peer Review File

Rapid Whole Genome Sequencing Enhances Pediatric Cancer Diagnosis

Corresponding Author: Professor David Rowitch

This manuscript has been previously reviewed at another journal. This document only contains information relating to versions considered at Nature Communications.

This file contains all reviewer reports in order by version, followed by all author rebuttals in order by version.

Version 0:

Reviewer comments:

Reviewer #5

(Remarks to the Author)
The authors claim in the title that rWGS improves clinical impact. However, this study does not have direct evidence to support this claim. There wasn't a head-to-head comparison of disease outcomes of patients for whom SOC WGS versus rWGS was used. There is potential to improve clinical impact based on indirect evidence, but this study does not provide direct evidence. The recommendation is to change the title so that it does not state definitively that it improves clinical impact.

* Please provide the standard deviations of the mean turnaround times that are reported. (abstract, line 36)

* What was the denominator for the 11% that is reported in line 39 of the abstract (see also later comment regarding this value).

* Please provide the 95% confidence interval for the estimate of change in clinical management for 18 (51%) of the patients (line 42 of the abstract).

* The authors frequently cite improvement in clinical impact. It is not clear that the use of rWGS would result in improved patient outcomes, which is typically what is meant by improvement in clinical impact. What is shown is the potential impact on clinical management decisions. It does not show that a change in clinical management resulted in improved patient outcome; this would require a different study design.

* The correlation reported in Figure 2A may be too good due to the influence of the two upper rightmost points. To remove the influence of these points on the correlation coefficient, please report the Spearman correlation.

* It is not clear why the denominator of 155 (number of variants identified by SOC WGS) is used to report the percent of variants identified by rWGS only (page 4, line 100). It does not make sense to use this as the denominator. The more appropriate denominator would be the total number of variants detected by either WGS and rWGS (so it would be larger than 155).

* Provide the standard deviations for the mean estimates of TAT (page 4, line 11).

* It is not clear if the subgroup means are for SOC WGS. If so, why aren't the rWGS means also provided (page 4, line 12)?

* In Supplementary Figure 1, what are the error bars? They should be the standard deviation (versus the standard error of the mean).

* What metric was used to establish equivalent detection across fresh and archival fresh frozen (pate 5, line 116)?

* Were the 8 variants detected by WGS and not rWGS clinically actionable (page 8, lines 125-130, these are not discussed).

* Given the 95% confidence interval for the estimate of 18/35 (page 5, line 120).

* The rWGS were run as part of this study and not part of a clinical workflow, and so, the turnaround times reported might be optimistic (i.e., this study only required analysis for 54 patients, of which 19 were retrospective. If they were implemented as part of clinical practice, it is unclear what impact this would have on turnaround times. Might be worth a discussion.

(Remarks on code availability)

Reviewer #6

(Remarks to the Author)
Thank you for your careful attention to the reviewers' comments and suggestions.

Additional suggested revisions:
In Figure 2B, it looks like there were 6 cases where rWGS showed unique variants; case 2, 8, 13, 29, 49, and 69. In Supplementary Table 2, when I sort for cases where the value of "detected by GMS-WGS" is "No", AND column N ("NHS SOC, including FISH, SNP, NGS, RNAfusion") is "No", 6 cases appear (2, 10, 11, 29, 49, 69). It seems like these six cases (2, 10, 11, 29, 49, 69) have variants (11 in total, variant ID's 6, 25, 33, 34, 83, 84, 85, 86, 87, 118, and 152) which were detected by rWGS but NOT confirmed by an orthogonal assay, and it seems like there is near or complete (if cases 10 and 11 are typos and meant to be the same cases as 8 and 13 are in Figure 2B) overlap of these cases with the cases highlighted in Figure 2B. Could the authors please clarify if/how these variants were confirmed prior to clinical implementation of the results? It seems like Supplementary Table 2 columns N & O address which orthogonal assays are included in NHS SOC, but do not help clarify how the findings which are unique to rWGS were confirmed.

There is a discrepancy between Supplementary Table 1 and Figure 1 (in Supplementary Table 1, patient 69 has an MMRD signature by GMS-WGS (and signature data was not available from rWGS), but that is not reflected in Figure 1A. Also, several cases do not have rWGS TMB data in Supplementary Table 1. Could the authors clarify why this analysis was not performed on some cases?

(Remarks on code availability)

Reviewer #7

(Remarks to the Author)
I co-reviewed this manuscript with one of the reviewers who provided the listed reports. This is part of the Nature Communications initiative to facilitate training in peer review and to provide appropriate recognition for Early Career Researchers who co-review manuscripts.

(Remarks on code availability)

Reviewer #8

(Remarks to the Author)
The authors evaluated a rapid WGS protocol using Illumina Constellation technology, which reduced the mean TAT from 44 days with standard NHS GMS-WGS to 3 days. The study included 54 children (35 prospective cases) with suspected pediatric cancer. rWGS detected 95% of clinically actionable variants identified by SOC testing and additionally identified 17 actionable variants not reported by SOC. Among the 35 prospective patients, rWGS informed a change in clinical management for half of the patients.

The substantially shortened TAT, improved variant detection enabled by enhanced coverage in difficult to map regions, and management improvements, highlight the clinical utility of rWGS in pediatric oncology.

In response to Reviewer 1's comments, the authors expanded their benchmarking beyond GMS-WGS alone to include SOC non-WGS molecular testing. They also provided illustrative case examples demonstrating the coverage advantages of Constellation technology in difficult-to-map genomic regions. In response to Reviewer 3's comments, the authors moderated their conclusions by clarifying that the study does not demonstrate that WGS outperforms SOC testing overall, but rather emphasizes the clinical impact of rapid turnaround time on patient care. They also further clarified the specific clinical benefits observed in selected cases. Overall, most of the key concerns raised by Reviewers 1 and 3 have been adequately addressed.

However, it remains unclear how Constellation - which provides long-range genomic data compared with conventional short-read sequencing, substantially enhances variant detection overall. While the authors presented two examples (PMS2 and ATXN1::NUTM2D) demonstrating improved coverage in difficult-to-map region; the 17 additional actionable variants identified by rWGS but not reported by SOC were largely attributable to differences in tissue sampling and pipeline limitations (Supplementary Table 2), rather than clear technological superiority in sequencing performance. Instead, the

manuscript primarily supports the benefit of improved TAT offered.
There is also some conceptual overlap in the claim of TAT improvements. The reported reduction in TAT appears partly driven by avoiding delays associated with standard workflows that can exceed six weeks due to technical and logistical requirements, such as fibroblast culture in hematological malignancies, as well as DNA extraction and library preparation. However, how the rWGS workflow compensates for bypassing fibroblast culture while still accurately identify somatic variants is not clearly described. It is also unclear which specific steps in logistics were optimized. For example, if standard WGS workflows were similarly streamlined through immediate library preparation and omission of fibroblast culture where feasible, turnaround times might also be significantly reduced. Without clearer methodological comparison, the magnitude of TAT improvement attributed specifically to Constellation technology may be somewhat overstated.

Nonetheless, the study demonstrates promising clinical potential, and continued accumulation of clinical cases is likely to further strengthen and refine this workflow for improving patient care.

(Remarks on code availability)

Version 1:

Reviewer comments:

Reviewer #5

(Remarks to the Author)
The authors were responsive to my suggestions. I have no additional suggestions.

(Remarks on code availability)

Reviewer #6

(Remarks to the Author)
Thank you for your careful attention to all of the reviewers' comments and suggestions.

Regarding the revisions related to reviewer #6's suggestion to revise Figure 2B: We thank the authors for clarifying their methodology for confirming variants which were uniquely identified by rWGS and making the aforementioned changes to the text. There still appear to be discrepancies between Figure 2B and Supplementary Table 2 (for example, in Supp. Table 2, patient 10 appears to have an NRAS variant which was only detected by rWGS, but this is not reflected in Figure 2B. Patients 15 and 17 also seem to have findings which were detected only by rWGS as indicated in Supp. Table 2, but Figure 2B shows them as blue, indicating that GMS and rWGS are concordant).

(Remarks on code availability)

Reviewer #7

(Remarks to the Author)
I co-reviewed this manuscript with one of the reviewers who provided the listed reports. This is part of the Nature Communications initiative to facilitate training in peer review and to provide appropriate recognition for Early Career Researchers who co-review manuscripts.

(Remarks on code availability)

Reviewer #8

(Remarks to the Author)
The revised text better acknowledges the limitations of the current study and appropriately moderates the claims regarding the technical advantages of the TruPath platform. I have no further questions.

(Remarks on code availability)

Open Access This Peer Review File is licensed under a Creative Commons Attribution 4.0 International License, which permits use, sharing, adaptation, distribution and reproduction in any medium or format, as long as you give appropriate credit to the original author(s) and the source, provide a link to the Creative Commons license, and indicate if changes were made.
In cases where reviewers are anonymous, credit should be given to 'Anonymous Referee' and the source.
The images or other third party material in this Peer Review File are included in the article's Creative Commons license, unless indicated otherwise in a credit line to the material. If material is not included in the article's Creative Commons license and your intended use is not permitted by statutory regulation or exceeds the permitted use, you will need to obtain permission directly from the copyright holder.
To view a copy of this license, visit https://creativecommons.org/licenses/by/4.0/

POINT-BY-POINT RESPONSES TO REVIEWER COMMENTS - NCOMMS-26-007421-T

Reviewer #5 (Remarks to the Author):

The authors claim in the title that rWGS improves clinical impact. However, this study does not have direct evidence to support this claim. There wasn't a head-to-head comparison of disease outcomes of patients for whom SOC WGS versus rWGS was used. There is potential to improve clinical impact based on indirect evidence, but this study does not provide direct evidence. The recommendation is to change the title so that it does not state definitively that it improves clinical impact.

Response: We have duly noted the reviewer's concern and comments in this and several other points below and have made numerous revisions. First, we have updated the title to: "Rapid Whole Genome Sequencing Enhances Pediatric Cancer Diagnosis". Second, we have updated the text throughout to reflect the fact that our study focused on diagnosis rather than providing direct evidence for improving clinical impact, and we have included a "Limitations" section after the Discussion (page 7).

1. Please provide the standard deviations of the mean turnaround times that are reported. (abstract, line 36)

Response: We have now included more precise language in the text; however, we have used simplified phraseology in the abstract.

The standard deviations of the mean TAT for each platform are:

- GMS WGS mean 42 days, SD 30
- rWGS mean 3.14 days, SD 1.24

This detail has been added to the manuscript text (page 4).

2. What was the denominator for the 11% that is reported in line 39 of the abstract (see also later comment regarding this value).

Response: The denominator for all calculations was 155 (total number of variants detected by either method; Suppl. Table 2). The abstract and text (page 4) have been revised to specify the denominator.

3. Please provide the 95% confidence interval for the estimate of change in clinical management for 18 (51%) of the patients (line 42 of the abstract).

Response: Apologies for the confusion. The prospective clinical-management figure has been corrected to 17/35 patients (49%). We now report this as a descriptive clinician-reported outcome rather than a formal estimate of patient outcome benefit (Discussion, page 6); hence the 95% confidence interval cannot be calculated.

4. The authors frequently cite improvement in clinical impact. It is not clear that the use of rWGS would result in improved patient outcomes, which is typically what is meant by improvement in clinical impact. What is shown is the potential impact on clinical management decisions. It does not show that a change in clinical management resulted in improved patient outcome; this would require a different study design.

Response: Please see response to point 1 above. We have now amended the manuscript throughout to reflect that the impact is on clinical diagnostics rather than outcome. Supplementary Table 4 has also been updated accordingly.

5. The correlation reported in Figure 2A may be too good due to the influence of the two upper rightmost points. To remove the influence of these points on the correlation coefficient, please report the Spearman correlation.

Response: The Spearman correlation is 0.77 and has been added to Fig 2A.

6. It is not clear why the denominator of 155 (number of variants identified by SOC WGS) is used to report the percent of variants identified by rWGS only (page 4, line 100). It does not make sense to use this as the denominator. The more appropriate denominator would be the total number of variants detected by either WGS and rWGS (so it would be larger than 155).

Response: We thank the reviewer for raising this point which was unclear. The 155 variants refers to the total detected by any method (rWGS and/or GMS WGS) (Suppl. Table 2). Please see response to point 2 above.

7. Provide the standard deviations for the mean estimates of TAT (page 4, line 11).

Response: Please see response to point 1 above. Supplementary Figure 1 has also been updated to represent standard deviations.

8. It is not clear if the subgroup means are for SOC WGS. If so, why aren't the rWGS means also provided (page 4, line 12)?

Response: These are for GMS-WGS (SOC). The rWGS subgroup means have also been added to this sentence now (page 5).

9. In Supplementary Figure 1, what are the error bars? They should be the standard deviation (versus the standard error of the mean).

Response: Please see response to point 7 above.

10. What metric was used to establish equivalent detection across fresh and archival fresh frozen (pate 5, line 116)?

Response: Equivalent variant detection was established by comparing SOC tests with rWGS in terms of clinically actionable variant calling. To clarify this in the manuscript, we have added a reference to Figure 1 and Supplementary Table 2, listing all the variants in detail.

11. Were the 8 variants detected by WGS and not rWGS clinically actionable (page 8, lines 125-130, these are not discussed).

Response: These were clinically actionable as defined by the NHS National Genomics Test Directory as reportable variants and the following clarification has been added to page 4.

"The eight variants not detected by rWGS were clinically actionable (as defined by the NHS National Genomics Test Directory), and discordance was attributed to tumour heterogeneity or low variant allele frequency (Figure 2C, Supplementary Table 3). Taken within the clinical context, the absence of these variants did not alter the clinical diagnosis or management of each individual patient in the study, as there were sufficient supporting variants to define the diagnosis in each case."

12. Given the 95% confidence interval for the estimate of 18/35 (page 5, line 120).

Response: Please see response to point 3 above.

* The rWGS were run as part of this study and not part of a clinical workflow, and so, the turnaround times reported might be optimistic (i.e., this study only required analysis for 54 patients, of which 19 were retrospective. If they were implemented as part of clinical practice, it is unclear what impact this would have on turnaround times. Might be worth a discussion.

Response: We thank the reviewer for this suggestion. A clarifying statement has been included in the Discussion (page 7) to address this, indicating that this is an area needing further research, which is in

progress.

Reviewer #6 (Remarks to the Author):

Thank you for your careful attention to the reviewers' comments and suggestions.

Additional suggested revisions:
In Figure 2B, it looks like there were 6 cases where rWGS showed unique variants; case 2, 8, 13, 29, 49, and 69. In Supplementary Table 2, when I sort for cases where the value of “detected by GMS-WGS” is “No”, AND column N (“NHS SOC, including FISH, SNP, NGS, RNAfusion”) is “No”, 6 cases appear (2, 10, 11, 29, 49, 69). It seems like these six cases (2, 10, 11, 29, 49, 69) have variants (11 in total, variant ID’s 6, 25, 33, 34, 83, 84, 85, 86, 87, 118, and 152) which were detected by rWGS but NOT confirmed by an orthogonal assay, and it seems like there is near or complete (if cases 10 and 11 are typos and meant to be the same cases as 8 and 13 are in Figure 2B) overlap of these cases with the cases highlighted in Figure 2B. Could the authors please clarify if/how these variants were confirmed prior to clinical implementation of the results? It seems like Supplementary Table 2 columns N & O address which orthogonal assays are included in NHS SOC, but do not help clarify how the findings which are unique to rWGS were confirmed.

Response: We thank the reviewer for requesting clarification. The variants uniquely identified by rWGS were validated by manual re-inspection of GMS WGS sequencing data using the integrated genomics viewer (IGV) of the relevant raw read alignments, the variants were not called in the GMS data due to bioinformatics analysis pipeline differences. We agree that manual re-interrogation of WGS data should not be described as orthogonal confirmation.

Actions: We have revised the manuscript and Supplementary Table 2 (column O) to distinguish between variants that were independently confirmed by an orthogonal clinical assay and variants that were verified by manual re-interrogation of GMS WGS sequencing data. The text has been revised to state that rWGS-specific findings were either confirmed by orthogonal assays where possible or verified by manual re-interrogation of the GMS WGS sequencing data (page 5). We have also clarified the case numbering discrepancy between Figure 2B and Supplementary Table 2.

There is a discrepancy between Supplementary Table 1 and Figure 1 (in Supplementary Table 1, patient 69 has an MMRD signature by GMS-WGS (and signature data was not available from rWGS), but that is not reflected in Figure 1A. Also, several cases do not have rWGS TMB data in Supplementary Table 1. Could the authors clarify why this analysis was not performed on some cases?

Response: Only germline sequencing was performed for Patient 69 through rWGS due to unavailability of sufficient tumour material, therefore somatic variant comparison, including mutational signature analysis was not performed. This has now been clarified in the Figure 1 legend and somatic GMS-WGS results have been removed from the plot. TMB comparison has only been performed for cases with both tumour and germline data available, due to limitations of TMB calculation in the tumour-only analysis setting. Therefore, TMB is not reported for a subset of cases (haematological malignancies). This has now been clarified in the supplementary methods section under “*Calculation of Tumor Mutational Burden (TMB) and Mutational Signatures”.*
We now clarify that patient 49 had a benign mass. Thus, both methods detected a TMB of 0 and Supplementary Table 2 has now been updated to reflect this.

Reviewer #7 (Remarks to the Author):

I co-reviewed this manuscript with one of the reviewers who provided the listed reports. This is part of the Nature Communications initiative to facilitate training in peer review and to provide appropriate recognition for Early Career Researchers who co-review manuscripts.

Response: We thank Reviewer #7 for their kind review and contribution.

Reviewer #8 (Remarks to the Author):

The authors evaluated a rapid WGS protocol using Illumina Constellation technology, which reduced the mean TAT from 44 days with standard NHS GMS-WGS to 3 days. The study included 54 children (35 prospective cases) with suspected pediatric cancer. rWGS detected 95% of clinically actionable variants identified by SOC testing and additionally identified 17 actionable variants not reported by SOC. Among the 35 prospective patients, rWGS informed a change in clinical management for half of the patients.

The substantially shortened TAT, improved variant detection enabled by enhanced coverage in difficult to map regions, and management improvements, highlight the clinical utility of rWGS in pediatric oncology.

In response to Reviewer 1's comments, the authors expanded their benchmarking beyond GMS-WGS alone to include SOC non-WGS molecular testing. They also provided illustrative case examples demonstrating the coverage advantages of Constellation technology in difficult-to-map genomic regions. In response to Reviewer 3's comments, the authors moderated their conclusions by clarifying that the study does not demonstrate that WGS outperforms SOC testing overall, but rather emphasizes the clinical impact of rapid turnaround time on patient care. They also further clarified the specific clinical benefits observed in selected cases. Overall, most of the key concerns raised by Reviewers 1 and 3 have been adequately addressed.

However, it remains unclear how Constellation - which provides long-range genomic data compared with conventional short-read sequencing, substantially enhances variant detection overall. While the authors presented two examples (PMS2 and ATXN1::NUTM2D) demonstrating improved coverage in difficult-to-map region; the 17 additional actionable variants identified by rWGS but not reported by SOC were largely attributable to differences in tissue sampling and pipeline limitations (Supplementary Table 2), rather than clear technological superiority in sequencing performance. Instead, the manuscript primarily supports the benefit of improved TAT offered.
There is also some conceptual overlap in the claim of TAT improvements. The reported reduction in TAT appears partly driven by avoiding delays associated with standard workflows that can exceed six weeks due to technical and logistical requirements, such as fibroblast culture in hematological malignancies, as well as DNA extraction and library preparation. However, how the rWGS workflow compensates for bypassing fibroblast culture while still accurately identify somatic variants is not clearly described. It is also unclear which specific steps in logistics were optimized. For example, if standard WGS workflows were similarly streamlined through immediate library preparation and omission of fibroblast culture where feasible, turnaround times might also be significantly reduced. Without clearer methodological comparison, the magnitude of TAT improvement attributed specifically to Constellation technology may be somewhat overstated.

Nonetheless, the study demonstrates promising clinical potential, and continued accumulation of clinical cases is likely to further strengthen and refine this workflow for improving patient care.

Response: We thank the reviewer for raising several important points.

1. Regarding the technical advantages of rWGS (TruPath) versus SOC short read WGS, this includes enhanced detection of structural variants and expansion repeats due to its improved longer read detection, as well as speed. Given the limited number of clinical cases analysed in this study, we cannot make definitive conclusions about technical superiority. Our findings do suggest that TruPath can close gaps in SOC WGS analysis including: (i) enabling germline variant phasing of distant variants (exemplified by Patient 69 with a diagnosis of

constitutional MMRD), improved genome coverage due to mapping improvements in repetitive regions (exemplified with NUTM2D gene coverage), as well as using proximity links to confirm large genomic rearrangements (exemplified by the NUTM2D::ATXN1 gene fusion). Future studies including larger number of cases should help establish the true value of TruPath technology in oncology applications.
We have revised the text to indicate the limitations of the study and potential technical advantages of rWGS that need further validation.

2. We agree with the reviewer that differences between SOC and rWGS TAT reflects in part the improved rWGS workflow, including logistical and technology contributions. By omitting the need for DNA extraction (in this study) and library preparation steps, TruPath technology is enabling even faster TAT with very much simpler workflow in comparison to standard WGS also reducing the expertise/ training needed to implement this approach.

3. Importantly, the rWGS workflow employed a tumour only approach for haematological malignancies to avoid the need for fibroblast cultures required for germline. The study supports the approach which will also be further evaluated in a follow-on implementation research study.
We have revised the text to indicate the limitations of the study and technical advantages of this workflow.

REVIEWERS' COMMENTS

Reviewer #6 (Remarks to the Author):

Thank you for your careful attention to all of the reviewers' comments and suggestions.

Regarding the revisions related to reviewer #6's suggestion to revise Figure 2B: We thank the authors for clarifying their methodology for confirming variants which were uniquely identified by rWGS and making the aforementioned changes to the text. There still appear to be discrepancies between Figure 2B and Supplementary Table 2 (for example, in Supp. Table 2, patient 10 appears to have an NRAS variant which was only detected by rWGS, but this is not reflected in Figure 2B. Patients 15 and 17 also seem to have findings which were detected only by rWGS as indicated in Supp. Table 2, but Figure 2B shows them as blue, indicating that GMS and rWGS are concordant).

Many thanks for noting this discrepancy, we have now updated Fig 2B to reflect the findings in Supplementary Table 2 for these patients.
